# Pharmacoepidemiology of common colds and upper respiratory tract infections in children and adolescents in Germany

**DOI:** 10.1186/2050-6511-15-44

**Published:** 2014-08-09

**Authors:** Nathalie Eckel, Giselle Sarganas, Ingrid-Katharina Wolf, Hildtraud Knopf

**Affiliations:** 1 German Institute of Human Nutrition Potsdam-Rehbrücke, Department of Molecular Epidemiology, Arthur-Scheunert-Allee 114-116, 14558 Nuthetal, Potsdam, Germany; 2 Robert Koch-Institute, Department of Epidemiology and Health Monitoring, General-Pape-Str. 62-66 12101, Berlin, Germany

**Keywords:** Common cold, Upper respiratory tract infection, Cough & cold medicines, Pharmacotherapy, Antibiotics, Children, KiGGS

## Abstract

**Background:**

Medicines to treat common colds (CC) and upper respiratory tract infections (URTI) are widely used among children, but there are only few data about treatments actually applied for these diseases. In the present study we analyze the prevalence and correlations of self-medicated and prescribed drug use for the treatment of CCs and URTIs among children and adolescents in Germany.

**Methods:**

Medicine use during the week preceding the interview was recorded among 17,450 children (0–17 years) who participated in the drug interview of the 2003–2006 German Health Interview and Examination Survey for Children and Adolescents (KiGGS). The definition of CCs and URTIs in the present study included the WHO-ICD-10 codes J00, J01.0, J01.9, J02.0, J02.9, J03.0, J03.9, J04.0, J06.8, J06.9, J11.1, J11.8, R05 and R07.0. Using the complex sample method, the prevalence and associated socio-demographic factors of self-medication, prescribed medicines and antibiotics were defined.

**Results:**

13.8% of the participating girls and boys use drugs to treat a CC or an URTI. About 50% of this group use prescribed medications. Among the users of prescribed medication, 11.5% use antibiotics for the treatment of these diseases. Looking at all prescribed medicines we find associations with younger age, immigration background, and lower social status. Antibiotic use in particular is associated with female sex, higher age, residency in the former East Germany and immigration background.

**Conclusions:**

The use of medicines to treat CCs or URTIs is widespread among children and adolescents in Germany. Thus, longitudinal studies should investigate the risks associated with this drug use. Differences in socio-demographic variables regarding exposure to antibiotic use indicate that there could be an implausible prescribing behavior among physicians in Germany.

## Background

Common colds (CCs) and other upper respiratory tract infections (URTIs) are usually self-limiting conditions with a high prevalence worldwide. Earlier analyses of the German Health Interview and Examination Survey for Children and Adolescents (KiGGS) indicate that the 1-year-average-prevalence of CCs among children and adolescents amounts to 88.5%, with the highest prevalence among children aged 3 to 6 years - almost 94% [1]. According to the literature, an average child undergoes a minimum of 4 to 8 URTIs per year [2-4]. Due to missing or low immunity in the first years of life, children are particularly vulnerable to viral infections [5].

Medicines against CCs and its symptoms are widely marketed, e.g. cough medicines, nasal decongestants, throat medicines, but also vitamins and herbal or homeopathic medicines. Data of the statutory health insurance document that 9 of the 20 most often prescribed drugs for children and adolescents belong to cough&cold medicines (CCMs) [6]. Additionally, many of these drugs are acquired over-the-counter (OTC) [7]. Analyzing children’s and adolescents’ use of medications to treat CCs and URTIs throws light on the prevalence and related factors of drug use and is an essential step for understanding issues concerning their safety. Data from health insurances and sales figures are not able to completely provide this transparency as they do not necessarily correspond to the actual medication usage. In the USA, serious adverse events and even some deaths are associated with the use of OTC CCMs [8,9]. About 7% of all pediatric prescriptions for the respiratory tract system are not officially licensed for use in children, which means that they have never been tested rigorously for pediatric safety and efficacy [10]. Earlier analyses of KiGGS suggest that about 30% of the medicines are used off-label in terms of under-dosing, over-dosing, untested indication, or age [11].

Antibiotics are usually not indicated for viral infections such as uncomplicated URTIs or CCs. Antibiotics do not lead to an improvement of CC and URTI symptoms, but they yield potential side effects [12,13]. Nevertheless, their use is common: Data from the statutory health insurance show that an URTI is the main reason for an antibiotic prescription. The data also indicate that antibiotic use is highest among children aged 0 to 4 years [14]. High and unnecessary antibiotic consumption is not only a problem for the individual but for the whole population, as this is one of the main reasons for antibiotic resistances [15].

However, national representative data regarding the pharmacoepidemiology of CCs and URTIs in the child population of Germany are lacking. By analyzing data of the KiGGS survey we attempt to fill this knowledge gap. We describe prevalence rates, investigate socio-demographic characteristics and analyze factors associated with the use of CC and URTI medicines. The analyses are differentiated into the use of overall CCMs, prescribed CCMs, self-medicated CCMs, and antibiotic use.

## Methods

KiGGS, a nationwide representative Health Interview and Examination Survey for children and adolescents, was conducted by the Robert Koch Institute between May 2003 and May 2006. The target population comprised all non-institutionalized residents of Germany between 0 and 17 years of age. Therefore, children and adolescents with a foreign nationality were also included. A detailed description of the methods of KiGGS have been published elsewhere [16]. Briefly, two-stage sampling procedures were applied. In the first stage 167 municipalities were drawn. This sample was representative for municipality sizes and structures in Germany. In the second stage, samples of children and adolescents aged between 0 and 17 years were drawn randomly from the corresponding local population registries. In total, 17,641 participants were included in the survey which equated to a response rate of 66.6%. Nonresponse analysis showed only little differences in socio-demographic and health-related variables between responders and non-responders. The survey was approved by the Ethics Committee of the Virchow Hospital, Humboldt University Berlin and federal data-protection officials. The children’s parents/guardians and/or children aged 14 years or older were informed about all aspects of the survey and they submitted a written consent [17].

Standardized, age-specified self-administered questionnaires filled out by parents/guardians and children aged 11 years or older were used to collect socio-demographic data, family background and health-related issues. The children’s age was categorized in 5 age groups: 0–2, 3–6, 7–10, 11–13, and 14–17 years. Children with immigration background were defined as those who had no German nationality themselves or whose parents had no German nationality. In order to calculate the socio-economic status according to Winkler, the parents’ education, professional classification and household net income were inquired [16]. Medicine use was investigated in a standardized computer-assisted face-to-face-interview by a physician. Information on medicine use was collected by asking the parents and the children themselves. The participants were asked to bring all of the original packages of medicines used in the last 7 days to the interview. Drug use was assessed by the following question: *“Has your child taken any medicines in the last 7 days? Please also mention any ointments, lineaments, contraceptive pills, vitamin and mineral supplements, medicinal teas, herbal medicines and homeopathic medicines.”* For all of the used drugs further information was collected, such as the form of administration, frequency of intake, origin (‘prescribed by a doctor’, ‘prescribed by a non-medical practitioner’, ‘bought over the counter’, ‘obtained from other sources’), duration of use, improvement of the condition(s) treated, and degree of tolerability. In addition, up to two conditions for which the medication was taken were registered. The reported medicines were classified according to the Anatomical Therapeutic Chemical (ATC) codes and the conditions treated according to the WHO International Classification of Diseases-10 codes (WHO ICD-10 codes) [18]. To evaluate the medication used for CCs or URTIs we included the following conditions (WHO-ICD-10 codes in brackets): CC (J00), acute sinusitis (J01.0, J01.9), acute pharyngitis (J02.0, J02.9), acute tonsillitis (J03.0, J03.9), acute laryngitis (J04.0), acute respiratory tract infections (J06.8, J06.9), influenza (J11.1, J11.8), cough (R05) and sore throat (R07.0).

All statistical analyses were performed using SPSS statistical software 18.0. The analyses were performed with a weighting factor to adjust for deviations in demographic characteristics (age, sex, residence in West or former East Germany, level of urbanity) in comparison to the national child population. Descriptive statistics were used to estimate the prevalence of medication use to treat CCs or URTIs according to sex, age, region of residence, immigration background, and social status. Prevalence of self-medication, prescribed medicines and antibiotic use were estimated among those children who used drugs to treat CCs or URTIs. Odds Ratios (ORs) were obtained from multivariate logistic regression models to depict associations between socio-demographic factors and self-medication, use of prescribed medicines and antibiotic use. Results with a probability level of *p* < 0.05 and 95% confidence intervals (CIs) not including the value 1 were considered as statistically significant.

## Results

**Figure 1:**
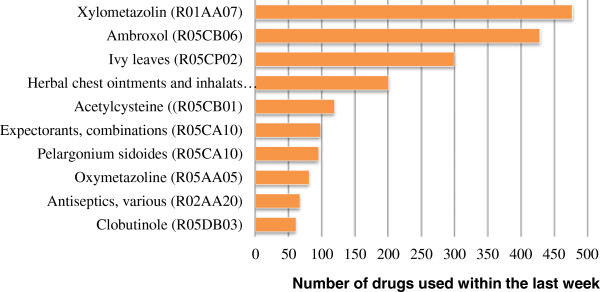
**The 10 most frequently used active ingredients to treat common colds (CCs) or upper respiratory tract infections (URTIs).** German Health Interview and Examination Survey for Children and Adolescents (KiGGS), 2003-2006.

Characteristics of the study population stratified by gender are listed in Table 1. 16.5% of all participants are living in the former East Germany and about 17% have an immigration background. The largest part of the boys and girls come from families with an intermediate social status. No significant differences are observed between girls and boys regarding the listed socio-demographic characteristics.2,595 children and adolescents used 3,648 medicines to treat a CC or URTI during the last 7 days. The vast majority of these medicines are drugs acting on the respiratory system (ATC code R00, 84.0%), followed by homeopathic medicines (Z00, 5.0%) and anti-infectives for systemic use (J00, 4.7%). 21% of the used medicines are not recorded on the last 5th level of the ATC-codes (level of the chemical substance), because participants forgot the brand names. Therefore, only 2,887 medicines could get analyzed on the basis of their active ingredients. The 10 most frequently used active ingredients are shown in Figure 1.

**Table 1:** Socio-demographic characteristics of survey participants by gender

	**Boys**		**Girls**	
	**n**	**% (95% CI)**	**n**	**% (95% CI)**
**Age (years)**				
0-2	1397	13.6 (13.2, 13.9)	1373	13.6 (13.2, 14.0)
3–6	1925	21.0 (20.7, 21.3)	1907	21.1 (20.8, 21.4)
7-10	2103	21.7 (21.4, 22.1)	2004	21.8 (21.4, 22.1)
11-13	1572	17.3 (17.0, 17.6)	1468	17.3 (17.0, 17.7)
14-17	1883	26.4 (25.8, 27.0)	1818	26.3 (25.7, 26.9)
**Region**				
East	2889	16.5 (12.3, 21.9)	2847	16.5 (12.3, 21.9)
West	5991	83.5 (78.1, 87.7)	5723	83.5 (78.1, 87.7)
**Urbanity**				
Rural town	1958	17.9 (12.6, 27.8)	1939	17.9 (12.6, 24.8)
Small town	2337	27.6 (20.9, 35.6)	2229	27.2 (20.5, 35.1)
Medium-sized town	2498	29.0 (22.2, 37.0)	2475	29.3 (22.4, 37.2)
Large city	2087	25.5 (25.5, 19.0)	1927	25.6 (19.1, 33.5)
**Immigration background**				
Yes	1350	17.4 (15.4, 19.6)	1230	16.9 (14.9, 19.1)
No	7498	82.6 (80.4, 84.6)	7592	83.1 (80.9, 85.1)
**Social class**				
Low	2454	27.7 (26.1, 29.4)	2306	27.3 (25.9, 28.8)
Intermediate	4011	45.2 (43.7, 46.8)	3890	45.7 (44.1, 47.2)
High	2185	27.0 (25.2, 29.0)	2181	27.1 (25.2, 29.0)
**Total**	8880	100	8570	100

n unweighted; % weighted.

German Health Interview and Examination Survey for Children and Adolescents (KiGGS), 2003–2006.

Table 2 illustrates the prevalence rates of drug use to treat CCs or URTIs during the last 7 days. 14.3% (95% CI 13.2%, 15.5%) of the study participants use medicines to treat a CC or an URTI. The use of those medicines decreases with rising age. All other socio-demographic variables (region, immigration background and social status) show no statistically significant differences. About half of the children using drugs to treat CCs or URTIs, utilize drugs prescribed by a physician (Table 2). The proportion decreases with rising age. Furthermore, we observed a statistically significant association between the use of prescribed medicine and immigration background as well as lower socio-economic status. The proportion of using self-medication to treat CCs and URTIs amounts to 57.6%. This proportion increases with higher age and is also higher for children without migration background and for children from families with a high social status (Table 2). Among those children and adolescents who use prescribed drugs, the antibiotic use is 11.6% (95% CI 9.8%, 13.7%). The antibiotic use in the descriptive analysis is significantly associated with immigration background and older age.

**Table 2:** Prevalence rates, proportions of overall prescribed medicines, proportion of self-medication and proportion of prescribed antibiotics to treat common colds (CCs) or upper respiratory tract infections (URTIs) (n = 17,450)

	**Prevalence rates**		**Proportions of prescribed medicine**		**Proportions of self-medication use**		**Proportions of prescribed antibiotic**	
	**n**	**% (95% CI)**	**n**	**% (95% CI)**	**n**	**% (95% CI)**	**n**	**% (95% CI)**
**Gender**								
Boys	1255	14.0 (12.7, 15.3)	655	50.3 (47.1, 53.6)	688	56.4 (53.1, 59.6)	69	9.5 (7.5, 12.0)
Girls	1340	14.7 (13.5, 16.1)	685	49.8 (46.1, 53.5)	772	58.8 (55.4, 62.1)	97	13.6 (10.8, 17.0)
**Age (years)**								
0 - 2	610	22.0 (19.9, 24.2)	416	66.1 (61.1, 70.7)	249	42.0 (37.1, 47.0)	32	6.1 (3.9, 9.5)
3 - 6	855	23.0 (20.9, 25.2)	473	55.4 (51.6, 59.1)	451	52.9 (48.8, 56.9)	53	11.0 (8.1, 14.7)
7 - 10	520	12.3 (10.8, 14.1)	223	42.4 (37.1, 47.8)	328	63.1 (58.6, 67.4)	29	9.8 (6.2, 15.0)
11 - 13	303	9.7 (8.3, 11.2)	118	38.9 (33.4, 44.7)	209	69.7 (63.5, 75.3)	24	18.9 (11.6, 29.1)
14 - 17	307	8.2 (7.1, 9.5)	110	34.2 (27.8, 41.1)	223	73.4 (67.3, 78.7)	28	24.8 (16.8, 35.0)
**Region**								
East	834	13.3 (11.6, 15.2)	439	52.6 (46.8, 58.4)	468	57.1 (52.3, 61.8)	70	15.0 (11.9, 18.6)
West	1761	14.5 (13.3, 15.9)	901	49.6 (46.7, 52.5)	992	57.6 (54.8, 60.5)	96	10.9 (8.9, 13.3)
**Immigration background**								
Yes	352	12.0 (11.2, 14.4)	225	63.0 (57.2, 68.4)	150	43.3 (38.3, 48.4)	39	18.2 (13.2, 24.5)
No	2229	14.6 (13.2, 15.5)	1108	47.7 (44.9, 50.5)	1305	60.2 (57.6, 62.9)	127	10.1 (8.3, 12.2)
**Social status**								
Low	683	13.8 (12.8, 15.6)	408	57.6 (53.4, 61.7)	319	48.0 (44.0, 52.1)	53	12.3 (9.4, 15.9)
Intermediate	1151	13.9 (12.7, 15.2)	582	48.8 (45.1, 52.5)	669	59.7 (56.2, 63.0)	76	13.0 (10.0, 16.7)
High	712	16.0 (14.4, 17.6)	315	43.6 (39.6, 47.7)	456	64.4 (60.3, 68.4)	31	8.0 (5.3, 11.9)
**Total**	2595	14.3 (13.2, 15.5)	1340	50.1 (47.5, 52.7)	1460	57.6 (55.0, 60.1)	166	11.6 (9.8, 13.7)

German Health Interview and Examination Survey for Children and Adolescents (KiGGS), 2003–2006.

Multivariate logistic regression models show that the use of self-medication is significantly associated with higher age (OR 2.40; 95% CI 1.87, 3.07), no immigration background (OR 1.64; 95% CI 1.28, 2.11) and high (OR 1.77; 95% CI 1.42, 2.20) or intermediate (OR 1.44; 95% CI 1.16, 1.82) social status. In contrast, use of prescribed medicines is significantly associated with immigration background (OR 1.60; 95% CI 1.21, 2.11), younger age (OR 2.27; 95% CI 1.81, 2.85) and lower socio-economic status (OR 1.58; 95% CI 1.25, 1.99). Antibiotic use is significantly associated with immigration background (OR 2.37; 95% CI 1.51, 3.73), female gender (OR 1.52, 95% CI 1.05, 2.18), older age (OR 1.45; 95% CI 1.01, 2.08) and residency in former East Germany (OR 1.67; 95% CI 1.19, 2.34) (Table 3).

**Table 3:** Socio-economic characteristics associated with the use of self-medication, prescribed medicine and antibiotics to treat common colds (CCs) and upper respiratory tract infections (URTIs) (n = 17,450)

	**Self-medication**^**1**^	**Prescribed medicine**^**1**^	**Antibiotic use**^**1**^
	**OR**	**OR**	**OR**
**Sex**			
Boys	Reference	Reference	Reference
Girls	1.04 (0.87, 1.25)	1.04 (0.86, 1.26)	1.52 (1.05, 2.18)
**Age (years)**			
0-10	Reference	2.27 (1.81, 2.85)	Reference
11-17	2.40 (1.87, 3.07)	Reference	1.45 (1.01, 2.08)
**Region**			
East	Reference	1.23 (0.94, 1.60)	1.67 (1.19, 2.34)
West	1.14 (0.90, 1.45)	Reference	Reference
**Immigration background**			
Yes	Reference	1.60 (1.21, 2.11)	2.37 (1.51, 3.73)
No	1.64 (1.28, 2.11)	Reference	Reference
**Social status**			
Low	Reference	1.58 (1.25, 1.99)	1.57 (0.93, 2.64)
Intermediate	1.44 (1.16, 1.80)	1.55 (0.98, 2.53)	1.55 (0.87, 2.75)
High	1.77 (1.42, 2.20)	Reference	Reference

Odds ratios and 95% confidence intervals were obtained from multivariate regression models ^1^includes WHO-ICD-10 codes J00, J01.0, J01.9, J02.0, J02.9, J03.0, J03.9, J04.0, J06.8, J06.9, J11.1, J11.8, R05 and R07.0.

German Health Interview and Examination Survey for Children and Adolescents (KiGGS), 2003–2006.

## Discussion

The present study documents a high prevalence of medicine use to treat CCs or URTIs among children and adolescents in Germany. About 14% of the boys and girls use at least one of these medicines within a given week. The most frequently used medicines are drugs acting on the respiratory system followed by homeopathic medicines and anti-infectives for systemic use. About half of the children with medicine use to treat CCs and URTIs use prescribed medicine. Almost 60% of children with CCMs use self-medication. Self-medication is associated with higher age, no immigration background, and high or intermediate social-status. In contrast, children of younger age, with an immigration background, and from families with low social-status use significantly more often prescribed drugs. Furthermore, antibiotic use is significantly associated with higher age, female sex, immigration background, and residency in former East Germany.

Data of medicine use based on treated conditions are sparse worldwide. The Slone Survey (1999–2006), a representative random-digit-dialing survey collecting data on medication use among the USA population during the last 7 days, observes a prevalence of children’ s exposure to CCMs of 10.1%. The definition CCMs includes all oral medications containing ≥ 1 antitussive, decongestant, expectorant or first-generation-antihistamine [19]. In KiGGS, expectorants are more frequently used than antitussives (6.7 vs. 1%), whereas in the Slone Survey, antitussives are more often used than expectorants (4.1 vs. 1.5%). Furthermore, there are differences regarding the active ingredients for certain medications, such as in antitussives and expectorants. The Slone Survey shows a high usage of dextromethorphan in antitussives and guafenisin in expectorants, whereas in our study, the most frequent active ingredients in expectorants are ambroxol, ivy leafs and acetylcystein and the most often used active ingredient in antitussives is clobutinol. The use of clobutinol has ceased since the year 2007 when all medicines containing clobutinol were withdrawn from the market. Data from a cohort study in South-West England, which were collected by self-administered questionnaires, yield exposure prevalences of 43.1% to CCMs, 5.0% to rhinologicals and 4.3% to throat medicines in the last 12 months among children aged up to 7.5 years [20]. However, comparability of these results with our prevalence rates (8.9% for CCMs, 5.9% for rhinologicals, 1.0% for throat medicines) is limited, mainly because of the difference in the reference periods. Longer observation periods lead to higher prevalence rates but also increase susceptibility for recall-bias. Despite of a much shorter observation period in our survey, the prevalence rate of rhinological use is higher in our study. This implies the probability of a higher 12 month prevalence in Germany compared to South-West England.

In our study self-medication for treating URTI is more common among children with higher age and among those without immigration background. These findings correspond to earlier analyses of KiGGS data looking at overall self-medication [7]. Moreover, our results suggest that self-medication is associated with a higher social-status. The same results are reported by a study looking at overall self-medication in Dutch adolescents [21]. In contrast to self-medication, the prevalence of using prescribed medicine is decreasing with higher age in our study, which is in accordance with results of the SLONE survey and a cohort study in three European countries [19,22]. Furthermore, in the present study the use of prescribed medication to treat CCs and URTIs is strongly associated with having an immigration background and a lower social-status. This finding is partly in line with earlier studies. A study in Poland observes positive associations between physician consultations and low school-leaving qualifications, as well as between use of OTC medicines and a high household income when treating respiratory tract infections in adults [23]. An Israeli cross-sectional study analyzes reasons why patients with flu-like symptoms consult a doctor. The reason “to get a prescription” is associated with low school-leaving qualifications, low income and unemployment [24]. However, Dutch secondary data analysis observes no association between use of prescription medicines and social-status by adolescents for all conditions [21].

Earlier findings looking at prevalence rates for antibiotic use among children in Europe and in the USA range from 31% to 38% [25-27]. However, these data refer to patients who were visiting a physician, thus comparability to our findings is limited. Our results suggest an increasing antibiotic use with higher age and with female sex. A previous study based on KiGGS data shows that children of younger age are more often exposed to antibiotic use compared to older children [28]. However, this study analyses the overall antibiotic use while the present study only looks at antibiotic use for treatment of CCs and URTIs. Our findings are probably influenced by not having included otitis media in the definition of URTIs, as the prevalence of otitis media is strongly decreasing with higher age [1]. Health insurance data on antibiotic use in Germany does not differentiate according to age or indication [14]. Gender differences concerning antibiotic use are already known: Data from the National Ambulatory Medical Care Survey from 1992 in the USA indicate a significant positive association between antibiotic consumption and female sex [29]. Moreover, findings from Abbas et al. suggest higher antibiotic use for girls compared to boys in all age groups except for 2-4-years old children [30]. Our results regarding regional differences are in line with the results of the EVA survey (*E*inflüsse auf die ärztliche *V*erschreibung von *A*ntibiotika in Deutschland) which investigates influences on prescribing patterns by physicians in Germany. This survey demonstrates that physicians in the eastern part of Germany prescribe antibiotics more frequently than in the western part [31]. Moreover, our results suggest an association between antibiotic use for CCs and URTIs and children with immigration background. The findings of earlier studies investigating this association are inconsistent. Neither a Swedish prospective cohort study nor a Norwegian survey observes an association between antibiotic consumption and immigration background [32,33]. A Cyprian cross-sectional-study finds a higher inappropriate antibiotic use by children with immigration background [34]. Furthermore, data of an Italian cohort-study suggests a significant higher antibiotic prescribing rate for URTIs for children with immigration background [27]. Altiner et al. report as a result of a qualitative study that physicians often tend to misinterpret the patients’ demands and often feel urged by the patients to give them an antibiotic prescription. This pressure is especially felt in consultations with Turkish immigrants [35,36].

A major strength of our study is the large number of population-representative data with a high response-rate including non-responder-analyses and quality assurances measures. The parents or the adolescents themselves were asked to bring the packages of the medicines used in the previous week to the interview. In contrast to health-insurance data, we analyze the medication actually used by children and adolescents, not only prescribed and potentially never used medicines. However, our study has some limitations. Although the personal interview was conducted by a physician, indications were only reported by the parents or adolescents and were not validated. Because of language difficulties and possible cultural differences in symptom reporting, this might result in more imprecisely measured data particularly among children with immigration background. Recall bias has to be considered which would result in underreporting. By limiting the observation period to 7 days prior to the interview we tried to minimize recall bias. Because of the cross-sectional design of the survey it is not possible to draw conclusions on the risks children and adolescents are to exposed to the used medicines. A longitudinal study is required to examine this. Furthermore, although all reported conditions were documented and confirmed by medical professionals, a standardized severity assessment was not carried out. Thus, we are not able to asses if the antibiotic prescribing was unnecessary.

## Conclusions

In summary, our study shows that the medicine use to treat CCs or URTIs is highly prevalent among children in Germany. Thus, longitudinal studies should investigate potential risks concerning this drug use. Furthermore, differences in socio-demographic variables, particularly sex, age, immigration background, and the difference between West and former East Germany, regarding antibiotic use indicate that there could be an implausible prescribing behavior among physicians in Germany. Thus, physicians should get trained to follow established guidelines when prescribing antibiotics for CCs and URTIs.

## Competing interests

The authors declare that they have no competing interests.

## Authors’ contributions

NE and HK coordinated the conceptualization and conduction of the project. NE performed the statistical analysis, wrote and finalized the manuscript. HK provided specific knowledge, assisted in analyzing the data and interpreting the results and contributed writing to the manuscript. GS assisted in analyzing the data and interpreting the results. IKW provided specific knowledge, assisted in interpreting the results and finalizing the manuscript. All authors read and approved the final manuscript.

## Pre-publication history

The pre-publication history for this paper can be accessed here:

http://www.biomedcentral.com/2050-6511/15/44/prepub
